# Cell-Free MicroRNA Expression Profiles in Malignant Effusion Associated with Patient Survival in Non-Small Cell Lung Cancer

**DOI:** 10.1371/journal.pone.0043268

**Published:** 2012-08-24

**Authors:** Tingting Wang, Mingming Lv, Sunan Shen, Sheng Zhou, Ping Wang, Yueqiu Chen, Baorui Liu, Like Yu, Yayi Hou

**Affiliations:** 1 Immunology and Reproduction Biology Lab, Medical School & State Key Laboratory of Pharmaceutical Biotechnology and Jiangsu Key Laboratory of Molecular Medicine, Nanjing University, Nanjing, Jiangsu, China; 2 Department of Oncology, Drum Tower Hospital Affiliated to Medical School of Nanjing University and Clinical Cancer Institute of Nanjing University, Nanjing, Jiangsu, China; 3 First Department of Respiratory Medicine, Nanjing Chest Hospital, Nanjing, Jiangsu, China; Roswell Park Cancer Institute, United States of America

## Abstract

**Objective:**

MicroRNAs (miRNAs) expression is altered in cancer cells, and miRNAs could serve as diagnostic and prognostic biomarker for cancer patients. This study was designed to analyze circulating miRNAs expression in the malignant pleural effusion (MPE) and their association with patient survival in non-small cell lung cancer (NSCLC).

**Methods:**

Pleural effusion from 184 patients with NSCLC and MPE were collected. MiRNA microarray and bioinformatics interpretation were used to evaluate miRNA expression profiles in 10 NSCLC patients with different survival prognosis. Associations were validated in 184 patients (randomly classified into training and validation set with equal number in each group) using quantitative RT-PCR. Risk scores were formulated based on the expression signature of miRNAs. Clinical data, such as patient survival, were collected for correlation analysis.

**Results:**

Thirty-three miRNAs were found to be altered more than two-fold by microarray in malignant effusions between longer-survival and shorter-survival groups, and levels of five miRNAs (miRNA-93, miRNA-100, miRNA-134, miRNA-151 and miRNA-345) were significantly associated with overall survival. High expression of miR-100 and low expression of miRNA-93, miRNA-134, miRNA-151 and miRNA-345 were associated with poor survival in both the training and validation cohort. Patients with high risk scores had overall poor survival compared to the patients with low risk scores. Risk score was an independent predictor of patient survival.

**Conclusions:**

Expression patterns of miRNAs are systematically altered in MPE of patient with NSCLC. The five miRNA signature from the effusion may serve as a predictor for the overall survival of patients with lung cancers.

## Introduction

Non-small cell lung cancer (NSCLC) is one of the most prominent causes of cancer death worldwide. Fifteen percent of lung cancer patients may have malignant pleural effusion (MPE) at the time of initial diagnosis and half develop pleural effusion in a later stage of the disease [1]. MPE is a poor prognostic sign for patients with NSCLC. Tumor spread via survival and proliferation of tumor cells in pleural effusion is an important route of metastasis and a frequent cause of morbidity in NSCLC. Despite advances in treatment modalities, the median survival is very short. The present standard treatment appears to be maximal safe evacuation of the pleural fluid followed by intravenous chemotherapy or intrapleural chemotherapy [2].However, it was found that not all patients were benefited from the addition of chemotherapy, especially in patient with short overall survival time. Therefore, prognostic assessment of the patient is essential for the choice of better therapeutic strategies. Hsu et al. proved that expression levels of angiogenetic biomarker were significantly correlated with patient survival and pleural effusion control [3]. In addition, recent molecular and genetic profiling studies could identify several markers and unique signatures as diagnostic and prognostic factors of NSCLC. These findings opened up possibilities for non-invasive cancer diagnosis and prediction. Some of the findings are on the verge of being translated into clinical use.

MicroRNAs (miRNAs) are 18- to 25-nucleotides, non-coding RNA molecules that regulate the expression of many genes. Since their discovery, miRNAs have been found to regulate a variety of cellular processes including apoptosis, differentiation and cell proliferation [4], [5]. Abnormal expressions of specific miRNAs are implicated in the pathogenesis of various human cancers, and miRNA expression profiling of human tumors has identified signatures associated with diagnosis, staging, progression, prognosis and response to treatment. The prognostic potential of miRNA has been demonstrated for chronic lymphocytic leukemia [6], lung cancer [7], [8], breast cancer [9], and neuroblastomas [10]. In lung cancer, high levels of pre-miR-155 and low levels of let-7 was reported to be correlated with poor prognosis [11], while miR-34a could be used as a prognostic marker of relapse in surgically resected NSCLC [12]. Recently, several reports suggest that cell-free circulating miRNAs are detectable in serum/plasma and the levels of tumor-derived miRNAs elevated in the patients with lung cancers [13], [14], which suggest that blood-based miRNAs could emerge as revolutionary sources of biomarker for lung cancer diagnosis and prognosis. However, a miRNA signature in MPE that can predict the clinical outcome in NSCLC patients had not been found so far.

In our previous studies, we have successfully isolated cell-free nucleic acid in malignant effusions and demonstrated that cell-free BIRC5 mRNA could be used as a potent diagnostic biomarker for MPE [15]. We also found that miR-24 and miR-30d were differently expressed in benign and malignant effusions, while the cell-free miR-152 may be used to predict the chemosensitivity to docetaxel [16], [17]. Given the prognostic potential for miRNAs in cancer, the aim of this study was to determine whether cell-free miRNA in pleural effusion could be used to predict survival time of patients with malignant pleural effusion secondary to NSCLC.

## Results

### Detection of effusion miRNAs and bioinformatics interpretation of functional miRNAs

All of the patients were followed up until September 16, 2011 or at the date of death. All 184 patients survived for a median of 6 months (range: 1–33 months). The overall cumulative proportion of patients surviving at 3 months was 0.70, at 6 months was 0.45, and at 12 months was 0.11.

To detect different miRNAs between longer-survival group and shorter-survival group, microarray was developed in 10 patients. As shown in Table 1, 5 patients in the longer-survival group and 5 patients in the shorter-survival group were exactly matched in the frequencies of histologic type and stage, while age, smoking status, and gender were not significantly different between the two groups. 113 miRNAs were detected in these 10 patients by miRNA microarray (File S1). Using class comparison analysis, 33 independent miRNAs were identified to be differentially expressed in two groups with fold changes >2 (Table S1). A volcano plot provided further information about the significance and magnitude of expressive alteration of these selected miRNAs (Figure S1), which was helpful in judging the most significant candidates for follow-up studies.

**Table 1:** Characteristics of study population.

Characteristic	Discovery cohort			Training cohort (n = 92)	Validation cohort (n = 92)	*P* *
	Long Survival Group (n = 5)	Short Survival Group (n = 5)	*P* *			
	No. (%)	No. (%)		No. (%)	No. (%)	
Sex			0.22			0.65
Female	2 (40)	0 (0)		55 (59.8)	52 (56.5)	
Male	3 (60)	5 (100)		37 (40.2)	40 (43.5)	
Age (years)			0.24			0.65
<60	3 (60)	1 (20)		38 (41.3)	41 (44.6)	
≥60	2 (40)	4 (80)		54 (58.7)	51 (55.4)	
Smoking Status		0.40			0.75	
Nonsmoker	3 (60)	2 (40)		59 (64.1)	61 (66.3)	
Ever-smoker	2 (40)	3 (60)		33 (35.9)	31 (33.7)	
Histology			1.00			0.20
Adenocarcinoma	5 (100)	5 (100)		86 (93.5)	88 (95.7)	
Others	0 (0)	0 (0)		6 (6.5)	4 (4.3)	
Stage			1.00			1.00
I, II and III	0 (0)	0 (0)		0 (0)	0 (0)	
IV	5 (100)	5 (100)		92 (100)	92 (100)	

* Two sided X^2^ test or Student *t* test.

David gene annotation was used to interpret the biological effect of miRNAs filtered by volcano plot. According to the results of data mining, 31 GOs were classified on the basis of these miRNA targets (Figure S2A). Another functional analysis of miRNAs by KEGG revealed that 9 signal transduction pathways were shown to participate in the different survival time of patients with lung cancer (Figure S2B). These results represent novel evidence for the regulatory effect of miRNAs on lung cancer via target genes and signaling pathway. Additionally, miRNA-mRNA network analysis integrated miRNAs and mRNA by outlining the interactions of miRNA and targeted genes (Figure S3). Taken these bioinformatics interpretations of functional miRNAs together, 5 miRNAs (miRNA-93, miRNA-100, miRNA-134, miRNA-151 and miRNA-345) were selected for further analysis.

### Association between the expression of miRNAs in effusion and patient survival

**Figure 1:**
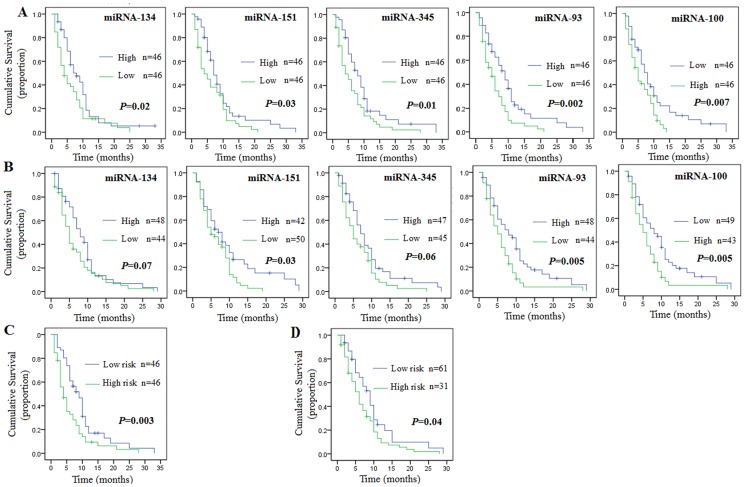
Kaplan-Meier survival estimates overall survival of lung cancer patients according to the miRNA expression signature. A) 92 patients in the training data set. B) 92 patients in the validation data set. C) Risk score and overall survival in training data set D) Risk score and overall survival in validation data set.

We then tested the predictive effects of these five miRNAs on lung cancer survival among 184 patients, randomly grouped into a training cohort (n = 92) and a validation cohort (n = 92). The characteristics of these patients with NSCLC were shown in Table 1. From the training cohort, high effusion expression levels of miR-100 (median, 0.02) and low expression levels of miR-134 (median, 0.50), miR-345 (median, 0.09) miR-151 (median, 0.05), and miR-93 (median, 0.03) were all individually associated with lower MSTs (7.1 months versus 4.3 months for miR-134, 7.1 months versus 3.9 months for miR-151, 8.2 months versus 2.9 months for miR-345, 9.2 months versus 5.5 months for miR-93, and 8.0 months versus 5.2 months for miR-100; Table 2 and Figure 1A). When these threshold values were applied to the validation set, comparable log-rank *P* values and HRs were observed (Table 2 and Figure 1B).

**Table 2:** Effusion expression levels of five miRNAs and survival of patients with lung cancers in training and validation cohort.

miRNA	Training cohort				Validation cohort			
	No. of patients	MST (months, 95% CI)		Log-rank *P*	No. of patients	MST (months, 95% CI)		Log-rank *P*
miR-134				0.02				0.07
>0.50	46	7.1	3.99–10.0		48	8.5	5.88–10.1	
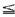 0.50	46	4.3	2.14–5.86		44	5.2	3.97–6.03	
miR-151				0.03				0.03
>0.05	46	7.1	5.57–8.43		42	7.1	4.48–9.53	
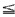 0.05	46	3.9	2.55–5.45		50	5.2	3.07–6.93	
miR-345				0.01				0.04
>0.09	46	8.2	5.92–10.1		47	7.2	5.23–8.77	
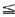 0.09	46	3.9	1.91–6.10		45	4.8	3.69–6.31	
miR-93				0.002				0.005
>0.03	46	9.2	6.53–11.5		48	8.4	4.10–11.9	
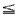 0.03	46	5.5	3.15–6.85		44	4.9	3.39–6.61	
miR-100				0.007				0.005
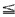 0.02	46	8.0	6.24–9.76		49	8.0	4.10–1.9	
>0.02	46	5.2	3.56–6.44		43	5.1	3.39–6.61	

Abbreviations: MST, median survival time;

Cox proportional hazard regression analysis for all the 184 samples showed that the expression levels of the five miRNAs were significantly associated with cancer death (*P* = 0.004 for miR-134, *P* = 0.01 for miR-151, *P* = 0.008 for miR-345, *P* = 0.03 for miR-93, and *P* = 0.02 for miR-100, Table 3). There were four miRNAs that were protective and one miRNA that was risky based on correlation of their expressions and association with patient survival.

**Table 3:** Cox regression analysis of five miRNAs for all the lung cancer patients with malignant effusions.

miRNA	Type	Hazard Ratio*	Coefficient	*P*
miR-134	protective	0.65	−0.28	0.004
miR-151	protective	0.66	−0.33	0.01
miR-345	protective	0.60	−0.35	0.008
miR-93	protective	0.69	−0.19	0.03
miR-100	risky	1.21	0.25	0.02

* Analysis was adjusted for age, gender, smoking status and histology.

### Nature of 5 miRNA expression signature

These 5 miRNAs were then used to create a signature by calculating a risk score for each patient. According to the risk scores, patients in the training cohort were divided into high and low risk groups by using the median risk score as cutoff. Patients belonging to high risk group had a shorter median survival than patients with low risk score (9.4 months versus 4.0 months, *P* = 0.003). The same risk-score formula and cutoff point obtained from the training cohort were applied to the 92 patients in the validation cohort. Similar to the findings from the training set, survival was greater in the low risk group than in the high risk group throughout the follow-up (Table 4, Figure 1C).

**Table 4:** Risk score and patient survival for training and validation cohort.

Risk score	MST (months, 95% CI)		Log-Rank *P*	Hazard Ratio* (95% CI)	
Training cohort			0.003		
Low risk (n = 46)	9.4	7.26–10.7		1.00	
High risk (n = 46)	4.0	2.59–5.41		2.50	1.40–3.85
Validation cohort			0.04		
Low risk (n = 61)	9.0	6.65–11.4		1.00	
High risk (n = 31)	5.8	4.68–7.32		1.62	1.11–2.84

* Analysis was adjusted for age, gender, smoking status and histology.

Abbreviations: MST, median survival time; CI, confidence interval.

### Effusion miRNA expression signature is independent of smoking status

In order to ascertain whether the 5 miRNA expression signature based risk score is an independent predictor of lung cancer patient's survival, we carried out Cox regression analysis. As shown in Table 5, smoking, age and risk score were statistically significant survival factors using a Cox univariate regression model. However, when a Cox multivariate regression model was applied, risk score (*P* = 0.01) and smoking (*P* = 0.04) were both independent predictors of patient survival (Table 5).

**Table 5:** Cox regression analysis of risk score and clinical characteristics in the entire patients (n = 184).

Variable	Hazard Ratio (95% CI)		*P*
Univariate analysis			
Risk score	2.35	1.73–4.21	0.005
Age	1.62	1.15–2.83	0.02
Histology type	0.86	0.36–2.05	0.75
Smoking status	2.16		0.009
Multivariate analysis			
Risk score	1.93	1.65–2.23	0.01
Age	1.25	1.08–1.42	0.07
Histology type	1.03	1.02–1.04	0.14
Smoking status	1.52	1.16–1.85	0.04

## Discussion

Using a sample-splitting approach, a 5 miRNA expression signature that can predict survival both in training and validation sets was identified in this study. More importantly, the 5 miRNA expression signature was an independent prognostic marker of poor survival time in multivariate analysis. To our knowledge, this is the first report of a miRNA expression signature in malignant pleural effusions that could predict NSCLC patient survival.

We used miRNA microarray to detect differently expressed miRNAs. Compared with shorter-survival group, 21 miRNAs were up-regulated and 12 miRNAs were down-regulated in longer-survival group. Most previous studies selected miRNAs based on the *P* values and Fold changes. Here, we additionally used bioinformatics interpretation of functional miRNAs to select miRNAs. GO term and KEGG pathway annotation were applied to their target gene pool. As a result, KEGG annotation showed that important proliferative (MAPK and Wnt), survival (TGF-β and mTOR), adhesive, oncogenic and metabolic signaling pathways were abundant among the significantly enriched ones. Most of them have already been reported to take part in lung cancer promotion. The GOs related to signal transduction, cell growth, apoptosis and metabolism represented most of the significantly enriched GO terms, which was in accordance with the KEGG analysis. This functional identity confirmed a systematic change in miRNAs and their target genes during tumor formation and promotion. The miRNA-mRNA interaction network analysis further integrated the bioinformatics finding, and then outlined the major targets of miRNAs. The center of the network was represents by degree, which means the contribution one miRNA to the genes around. miRNAs with higher degrees were selected for further analysis. Therefore, bioinformatics interpretation may help us pick up miRNAs with important functions during lung cancer progression.

The five miRNA signature identified in this study included one miRNA (miRNA-100) that was risky and four miRNAs (miRNA-93, miRNA-134, miRNA-151 and miRNA-345) that were protective with respect to their association between their expression and patient survival. The protective and risky nature of these miRNAs is suggestive of their functions being either inhibitory or promoting of various properties of cancer cells such as proliferation, migration and invasion. Increased miR-100 has been found in ovarian carcinoma [18], hepatocellular carcinoma and esophageal squamous cell carcinoma [19], [20]. In good correlation with our data, miR-100 expression was associated with progression and prognosis in gastric cancer [21]. Zheng et al. demonstrated that miR-100 regulates cell differentiation and survival by targeting RBSP3, a phosphatase-like tumor suppressor in acute myeloid leukemia [22]. Among the four protective miRNAs, only miR-134 was reported to increase the cell survival by inducing G1 arrest in lung cancer cells [23]. Our results show that all these four miRNAs were up-regulated in patients with longer survival time. The expressions of these miRNAs were positively correlated with patient survival. Infiltration of the effusion by lumophcytes may lead to a tumor immune response. Therefore, we speculate that the miRNAs associated with improved survival may indicate an inflammatory response, which needs to be proved.

In this study, cell-free miRNA profiles in pleural effusions were detected for the first time, which has a lot of advantages. For NSCLC patients with MPE, the goals of treatment are to control the volume of pleural effusion and prolong survival. Therefore, thoracoscopically evacuation of effusion is a routine in clinical. Collection of effusion sample did not increase burden on patients. Besides, patients with lung cancer and malignant effusions cannot receive surgical treatment. Effusion sample is easier to obtain than tissue sample. Moreover, miRNAs as biomarkers used for prognosis in clinical setting have important advantages in contrast to mRNAs. Unlike mRNAs, miRNAs in effusions remain largely intact and stable. A modest number of miRNAs may be sufficient to predict patient survival. It has been shown that classification of multiple cancers based on miRNA expression signatures is more accurate than mRNA based signatures [24].

Although further prospective studies with large numbers of patients are warranted to confirm the role of miRNA signature as a prognostic factor, the present results may have significant clinical implications, since there is no established prognostic marker except pretreatment performance status for NSCLC patients with MPE [25]. The five miRNA signature, identified in this study, classifies patients successfully into low risk and high risk groups in both training and validation sets. This may help clinicians to identify patients belonging to high risk for more effective adjuvant therapy in addition to the standard treatment protocol. Our finding that five miRNA signature can predict patient's survival also likely to generate potential molecular targets for the development of anticancer therapy. Since miRNAs can target multiple genes, more thorough studies are needed to understand the regulatory mechanism of these miRNAs which is likely to result in better understanding on pleural metastasis of NSCLC.

In conclusion, we have identified a five miRNA signature that can predict patient survival in advanced NSCLC. Effusions with high expression of miR-100 and low expression of miRNA-93, miRNA-134, miRNA-151 and miRNA-345 were associated with poor survival outcome. These findings may have implications in the understanding of advanced NSCLC, development of targeted therapy and selection of cancer patients for adjuvant therapy.

## Methods

All research involving human participants have been approved by the “Drum Tower Hospital ethics committee” and written informed consent was obtained from all subjects.

### Sample collection and RNA isolation

Effusion samples of 184 consecutive patients with histologically confirmed lung cancer were collected during the period March 2006 to December 2010 at Drum Tower Hospital and Nanjing Chest Hospital. None of the patients received cavity medicine delivery before sample collection. 5 ml effusion for RNA extraction was collected in a RNase-free tube containing citric acid from each patient. The supernatant was obtained by centrifugation at 4000 g for 20 min. 800 µl supernatant was mixed with 2.4 ml of TRIzol LS reagent (Invitrogen, CA, USA). Another 30 fmol ath-miR159a was supplemented into each sample tube. Next, total RNA containing small RNA was extracted from effusions using miRNeasy Mini Kit (Qiagen, Hilden, Germany) according to the manufacturer's protocol.

### Microarray and bioinformatics interpretation of functional miRNAs

MiRNA microarray was developed in 10 samples (Applied Biosystems, Foster City, CA, USA). After the significant analysis and FDR analysis, we selected the differentially expressed genes according to the *P*-value threshold and fold changes [26]. GO analysis was applied to analyze the main function of the differential expression genes according to the Gene Ontology which is the key functional classification of NCBI [27]. Similarly, Pathway analysis was used to find out the significant pathway of the differential genes according to KEGG, Biocarta and Reatome [28]. The relationship of the miRNA and genes were counted by their differential expression values, and miRNA -Gene-Network was built according to the interactions of miRNA and genes in Sanger miRNA database [29]. The center of the network was represents by degree, which means the contribution one MicroRNA to the genes around. The 5 miRNAs with highest degrees were chosen for further study.

### Quantitative RT-PCR

miRNAs were reverse-transcribed to cDNA with AMV reverse transcriptase (Takara, Japan). The total RNA (1.5 µL for each miRNA) was used as the templates, and the reaction was incubated at 16°C for 10 min, followed by 42°C for 40 min and 70°C for 15 min to denature the reverse transcriptase. Quantitative PCR reactions for miRNAs were carried out using UPL probe (Roche, Basel, Switerland) by ABI Stepone Plus Detection System (Applied Biosystems, Foster City, CA, USA). The PCR conditions were 95°C for 10 min, followed by 50 cycles at 95°C for 15 s and 60°C for 1 min. Each sample was assayed in triplicates. Relative miRNA expression levels were calculated according to the comparative Ct method using ath-miR159a as reference and a mixture of RNA extracted from human lung cancer A549 cells and gastric cancer BGC-823 cells (1∶1) as the calibrator.

### Statistical analysis

Differences between two groups were evaluated by the t-test. The associations between overall survival and effusion miRNA expression levels were analyzed by using the Kaplan-Meier method, log-rank test, and Cox proportional hazard regression models. Risk scores were assigned to all the patients according to a linear combination of the expression level of the miRNA, weighted by the regression coefficient from the training samples. The risk score was calculated as follows: risk-score = (1.21×expression level of miR-100)+(−0.28×expression level of miR-134)+(−0.33×expression level of miR-151)+(−0.35×expression level of miR-345)+(−0.19×expression level of miR-93). Cox stepwise regression model and stratification analyses also were conducted. Statistical analysis was performed using the software (SPSS 18; SPSS; Chicago, IL). Statistical significance was accepted for *P* values<0.05.

## Supporting Information

File S1 **113 miRNAs were detected in these 10 patients between longer-survival group and shorter-survival group by miRNA microarray (File S1).** Fold changes, FDR value and p-value were calculated for all miRNAs.
(XLS)

Figure S1 **The volcano plot shows the differentially expressed miRNAs in effusions between longer-survival group and shorter-survival group.** The horizontal axis represents the fold change between two groups. The vertical axis represents the P-value of the t-test for the differences between samples.
(TIF)

Figure S2 **GO and pathway analysis based on miRNA targeted genes.** The vertical axis is the GO (A) and pathway (B) category, and the horizontal axis is the enrichment of GO and pathways.
(TIF)

Figure S3 **miRNA–mRNA network. Red box nodes represent miRNA and blue cycle nodes represent mRNA.** Edges show the inhibitory effect of miRNA on mRNA. The center of the network was represents by degree, which means the contribution one MicroRNA to the genes around. miRNA-93 have the biggest degrees.
(TIF)

Table S1 **MiRNAs that are differentially expressed in effusions between longer-survival group and shorter-survival group.**
(DOC)
