# Supplementary figures and images for: Cell-Free MicroRNA Expression Profiles in Malignant Effusion Associated with Patient Survival in Non-Small Cell Lung Cancer
Source: PLoS One. 2012 Aug 24;7(8):e43268. doi: 10.1371/journal.pone.0043268 (PMC3427341; doi:10.1371/journal.pone.0043268)

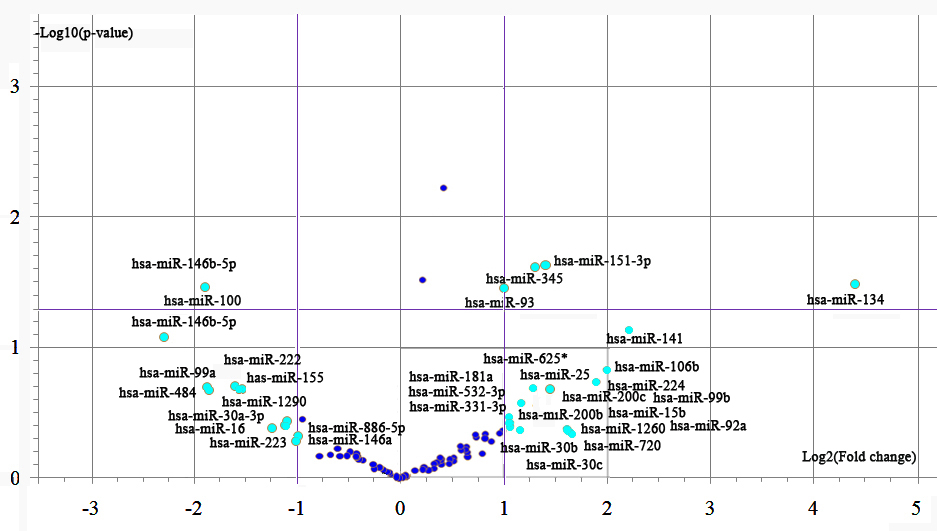

Supplement: Figure S1 — The volcano plot shows the differentially expressed miRNAs in effusions between longer-survival group and shorter-survival group. The horizontal axis represents the fold change between two groups. The vertical axis represents the P-value of the t-test for the differences between samples. (TIF) [file pone.0043268.s002.tif]

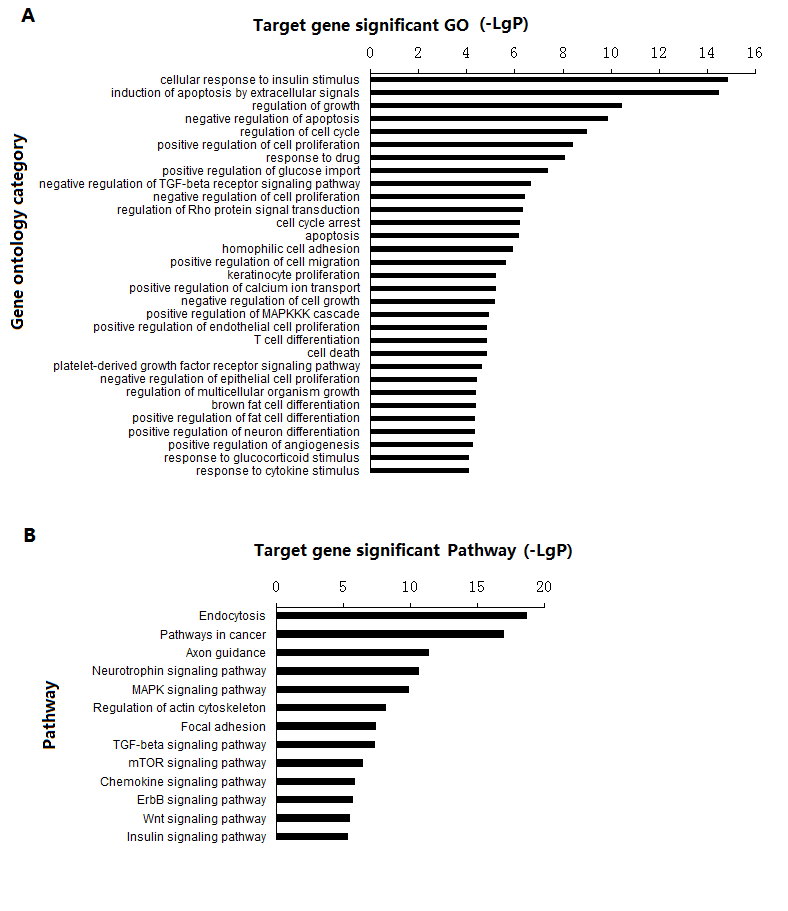

Supplement: Figure S2 — GO and pathway analysis based on miRNA targeted genes. The vertical axis is the GO (A) and pathway (B) category, and the horizontal axis is the enrichment of GO and pathways. (TIF) [file pone.0043268.s003.tif]

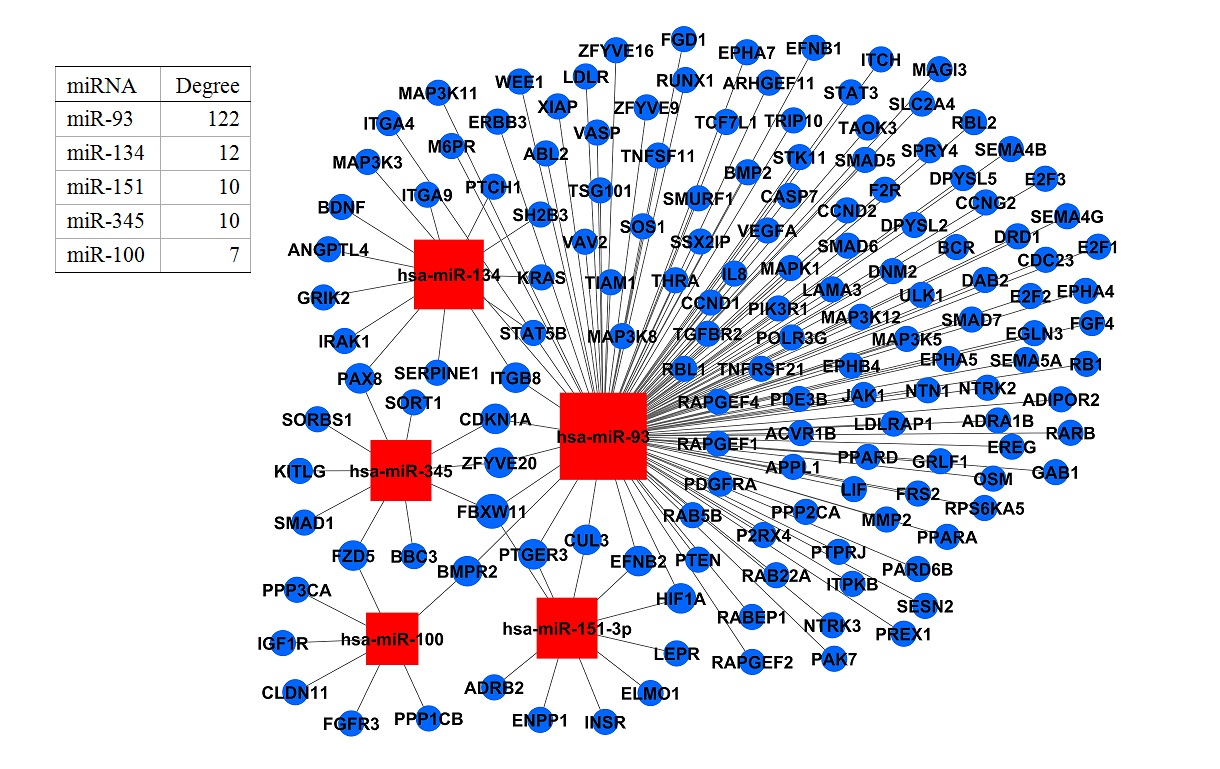

Supplement: Figure S3 — miRNA–mRNA network. Red box nodes represent miRNA and blue cycle nodes represent mRNA. Edges show the inhibitory effect of miRNA on mRNA. The center of the network was represents by degree, which means the contribution one MicroRNA to the genes around. miRNA-93 have the biggest degrees. (TIF) [file pone.0043268.s004.tif]
