# Supplementary material for: Cell-Free MicroRNA Expression Profiles in Malignant Effusion Associated with Patient Survival in Non-Small Cell Lung Cancer
Source: PLoS One. 2012 Aug 24;7(8):e43268. doi: 10.1371/journal.pone.0043268 (PMC3427341; doi:10.1371/journal.pone.0043268)
Supplement: Table S1 — MiRNAs that are differentially expressed in effusions between longer-survival group and shorter-survival group. (DOC) [file pone.0043268.s005.doc]

Table S1. MiRNAs that are differentially expressed in effusions between longer-survival group and shorter-survival group.

| Probe from microarray | *P* value ^a^ | FDR value ^b^ | Fold Change |
| --- | --- | --- | --- |
| MiRNAs with higher expression in longer-survival group | | | |
| hsa-miR-134 | 0.04 | 0.03 | 21.11 |
| hsa-miR-141 | 0.08 | 0.98 | 5.06 |
| hsa-miR-106b | 0.15 | 0.98 | 3.98 |
| hsa-miR-224 | 0.18 | 0.98 | 3.71 |
| hsa-miR-720 | 0.44 | 0.98 | 3.07 |
| hsa-miR-1260 | 0.42 | 0.98 | 3.05 |
| hsa-miR-200c | 0.19 | 0.98 | 2.70 |
| hsa-miR-92a | 0.37 | 0.98 | 2.64 |
| hsa-miR-151-3p | 0.03 | 0.03 | 2.64 |
| hsa-miR-99b | 0.37 | 0.98 | 2.63 |
| hsa-miR-15b | 0.26 | 0.98 | 2.54 |
| hsa-miR-30c | 0.35 | 0.98 | 2.51 |
| hsa-miR-25 | 0.28 | 0.98 | 2.49 |
| hsa-miR-345 | 0.03 | 0.03 | 2.46 |
| hsa-miR-625 | 0.20 | 0.98 | 2.43 |
| hsa-miR-200b | 0.26 | 0.98 | 2.25 |
| hsa-miR-30b | 0.43 | 0.98 | 2.22 |
| hsa-miR-331-3p | 0.38 | 0.98 | 2.08 |
| hsa-miR-532-3p | 0.40 | 0.98 | 2.08 |
| hsa-miR-181a | 0.35 | 0.98 | 2.06 |
| hsa-miR-93 | 0.04 | 0.03 | 2.00 |
| MiRNAs with reduced expression in longer-survival group | | | |
| hsa-miR-146a | 0.47 | 0.98 | 0.50 |
| hsa-miR-886-5p | 0.54 | 0.98 | 0.49 |
| hsa-miR-16 | 0.36 | 0.98 | 0.47 |
| hsa-miR-30a-3p | 0.39 | 0.98 | 0.47 |
| hsa-miR-223 | 0.43 | 0.98 | 0.42 |
| hsa-miR-1290 | 0.21 | 0.98 | 0.34 |
| has-miR-155 | 0.19 | 0.98 | 0.33 |
| hsa-miR-222 | 0.14 | 0.98 | 0.31 |
| hsa-miR-484 | 0.16 | 0.98 | 0.30 |
| hsa-miR-99a | 0.20 | 0.98 | 0.27 |
| hsa-miR-100 | 0.04 | 0.03 | 0.27 |
| hsa-miR-146b-5p | 0.07 | 0.98 | 0.21 |

a *P* values reported are the comparison analysis of differentially expressed miRNA from 10 malignant effusions using RVM t-test.

b FDR = False discovery rate. FDR is calculated by BRB array tools.
